# Supplementary material for: An adapted health system access framework for avoidable inflammatory bowel disease admissions: a scoping review
Source: Crohns Colitis 360. 2026 Jul 21;8(3):otag083. doi: 10.1093/crocol/otag083 (PMC13449608; doi:10.1093/crocol/otag083)
Supplement: Supplementary file 1 [file otag083_supplementary_data.docx]

**Supplementary A: Search strategy**

**Medline via Ovid**

1 - exp Inflammatory Bowel Diseases/

2 - enteritis/

3 - exp Crohns disease/

4 - exp ulcerative colitis/

5 - ("cleron disease" or "colitis ulcerativa" or "colitis ulcerosa" or "crohn disease*" or "crohns disease*" or enteritis or "inflammatory bowel disease*" or "inflammatory enteropath*" or "morbus crohn" or "mucosal colitis" or "regional enterocolitis" or "ulcerative colitis" or "ulcerative colorectitis" or "ulcerative procto colitis" or "ulcerative proctocolitis" or "ulcerous colitis").ti,ab,hw,kw.

6 - 1 or 2 or 3 or 4 or 5

7 - Avoidable.ab,ti

8 - Preventable.ab,ti

9 - Unplanned.ab,ti

10 - Unnecessary.ab,ti

11 - Unwarranted.ab,ti

12 - 7 or 8 or 9 or 10 or 11

13 - 6 and 12

14 - limit 22 to yr="2000 -Current"

**EMBASE via Ovid**

1 - exp Inflammatory Bowel Diseases/

2 - enteritis/

3 - exp Crohns disease/

4 - exp ulcerative colitis/

5 - ("cleron disease" or "colitis ulcerativa" or "colitis ulcerosa" or "crohn disease*" or "crohns disease*" or enteritis or "inflammatory bowel disease*" or "inflammatory enteropath*" or "morbus crohn" or "mucosal colitis" or "regional enterocolitis" or "ulcerative colitis" or "ulcerative colorectitis" or "ulcerative procto colitis" or "ulcerative proctocolitis" or "ulcerous colitis").ti,ab,hw,kw.

6 - 1 or 2 or 3 or 4 or 5

7 - Avoidable.ab,ti

8 - Preventable.ab,ti

9 - Unplanned.ab,ti

10 - Unnecessary.ab,ti

11 - Unwarranted.ab,ti

12 - ((unscheduled or unplanned or un-planned or unanticipated or unexpected) adj5 (admission* or readmission* or hospitali#ation or care)).mp.

13 - 7 or 8 or 9 or 10 or 11 or 12

14 - 6 and 13

15 - *Hospitalization/

16 - exp patient admission/

17 - *emergency/

18 - (hospital admission* adj5 emergenc*).mp.

19 - emergency medical admission*.mp.

20 - (hospital* adj5 readmission).mp.

21 - 15 or 16 or 17 or 18 or 19 or 20

22 - 14 and 21

23 - limit 22 to yr="2000 -Current"

**CINAHL**

inflammatory bowel disease or ibd or ulcerative colitis or crohn's disease

AND

avoidable hospital admissions or preventable hospital admissions or hospitalization or hospitalisation or hospital stay

Limiters: 2000-2024

**Supplementary file B, Table A**

*Supplementary Table A. Concepts of IBD admissions, qualitative descriptions and coding*

| **Author(s), year, country of study** | **Concept(s) defined/ explored within study article** | **Description and/or definition** | **Initial inductive codes applied to extract** |
| --- | --- | --- | --- |
| Melmed et al. (2021), USA | Unplanned urgent care | Pg2 “Quality of care for IBD can be deﬁned and measured using a framework of structure, process, and outcomes. Signiﬁcant variation in processes and outcomes of care exists in IBD, suggesting opportunities to improve care quality. Quality outcome measures for IBD include unplanned urgent care, hospitalizations, corticosteroid use, and opioid use.” | Suboptimal quality of care  Opportunity for quality improvement |
|  | Reduced ED visits and hospitalisations | Pg8 “Rapid access clinics have recently been shown to reduce ED visits and hospitalizations among those with IBD.” | Rapid access to outpatient care |
| Nene et al. (2020), Canada | Reduce ED visits | Pg761 “A large proportion of patients with known chronic  conditions could potentially be managed in alternative care settings, more specific to their disease. These specific “rapid access” patient pathways for “rapid” evaluation and management can be established through regular outpatient care providers, which can potentially reduce ED visits” | Access issues  Timely access to clinical evaluation & treatment  Rapid access to outpatient care |
|  | Avoiding ED visits | Pg761 “A rapid access clinic service can provide quick access and rapid evaluation by an IBD specialist for patients experiencing moderate to severe symptoms in non-emergency situations related to IBD, thus potentially avoiding ED visits.” | Rapid access  Timely access to clinical evaluation & treatment |
|  | Preventing unnecessary ED visits | …optimizing resource utilization for patient evaluation and treatment decisions to IBD specific medical care in urgent situations, thus preventing unnecessary ED visits.” | Optimising resources for patient evaluation and treatment decisions |
| Regueiro et al. (2018), USA | Reduction in unplanned care | Pg1873 “The reduction in unplanned care and QoL improvement was owing to 3 core elements: (1) stratiﬁcation of the determinants that drove health utilization,  (2) care plans that used the multidisciplinary team and (3) the partnership between the provider group and health plan around the collective care of a population of patients.” | Risk-stratified care  Use of patient care plans (strategies)  Collaborative health professional working |
| Malhotra et al. (2018), USA | Avoidable readmissions | Pg1 “Hospital readmission rate has been proposed as an important quality metric because such admissions are prevalent, expensive and potentially avoidable...  Readmitted patients are 55% more likely to have a quality-of-care problem such as inadequate discharge instructions or outpatient follow-up scheduling…  and repeat hospitalizations can be reduced by the intensive discharge planning of high-risk patients ...Consequently, it is imperative that organizations examine and implement strategies to avert avoidable readmissions.” | Suboptimal quality of care  Inadequate outpatient follow up  Discharge planning (strategies) |
|  |  |  |  |
|  | Reducing readmission |  |  |
|  |  | Pg3 “Various studies conducted on reducing readmission rates  in patients with chronic diseases suggest that closer outpatient follow-up may reduce rehospitalizations. Our study also showed that an outpatient visit was associated with reduced readmission rates.” | Close outpatient follow up |
|  |  |  |  |
|  |  |  |  |
| Coenen et al. (2017), Belgium | Reduce ER visits | Pg646 “In this way, the IBD nurse can provide better compliance, more early intervention during disease ﬂares, and as a consequence, reduce the number of outpatient visits and emergency room (ER) visits.” | Timely access to clinical evaluation & treatment  Early intervention during flare |
|  | Avoidance of ER visits | Pg647 “Avoidance of ER visit: by planning an urgent outpatient clinic visit at the gastroenterology or the abdominal surgery unit (<1 week from contact)...  planning an urgent endoscopic or radiological examination (<1 week from contact), direct admission to ward bed.” | Rapid access to outpatient care  Timely access to clinical evaluation & treatment |
| Zhen et al. (2021), Canada | Avoid urgent care and hospitalisations | Pg 2 “To avoid urgent care, hospitalisations and surgery, proactive care through ‘Treat to Target’ strategies aimed at the normalisation of patients symptoms and inflammatory markers are now considered best practice” | Proactive care (strategies) |
| Liu et al. (2019), USA | Reducing hospitalisations | Pg 1812-1813 “Similar to previous studies of PCMH care models, we found the emphasis on patient-centered care reduced IBD-related hospital stays by 18% in the first year.” | Patient-centred care approaches (strategies) |
| de Jong et al. (2017), Netherlands | Prevent admissions | Pg960 “Our findings accord with those in other chronic relapsing-remitting diseases and suggest that tight disease monitoring…  and early intervention in case of a relapse can prevent admission to hospital.” | Tight disease monitoring  Early intervention |
| Harvey et al. (2021), Canada | Without unplanned hospital attendance | Pg501 “The role of the IBD CNS team can also be explained  if required, along with details of the helpline, including how the patient can access help if their symptoms return. Should patients find that their symptoms are deteriorating, they then have access to the CNS team so that treatment can be escalated rapidly without the involvement of primary care teams or unplanned hospital attendances.” | Patient knowledge of how to access  Timely access to clinical evaluation & treatment |
|  | Reduction in admissions | Pg503 “It is plausible that the early evaluation call led to a reduction in admissions in the following 6 months. A combination of ensuring a smooth transition to maintenance therapy;... | Timely (early) access to clinical evaluation & treatment |
|  | Admission avoidance | a helpline for patients to call rather than present to the hospital or their GP; and early identification of patients who are not responding to corticosteroids; all likely contributed to medium-term admission avoidance” | Rapid access to clinical advice  Early intervention |
| Leach et al. (2014), Australia | Reducing hospital admissions | Pg371 “Advantages accruing from the IBD Nurse position relate in part to rapid access to advice, counselling and support via a single fixed “first point of contact” for patients, their families, GPs and non-IBD external gastroenterologists. At the most basic level this can be seen to facilitate prompt recognition of symptoms and early intervention during disease flares with consequent reduction in hospital admissions and outpatient appointments.” | Rapid access to clinical advice  Timely access to clinical evaluation & treatment  Early intervention during flare |
|  | Hospital admission avoidance | Pg371 “Admission avoided: patient managed successfully at home with close follow-up, instead of requiring inpatient care. Patient assessed by IBD Nurse as having moderately severe symptoms warranting admission with any further deterioration.” | Tight disease monitoring  Timely access to clinical evaluation & treatment |
|  | Emergency department avoidance | Pg371 “Streamlined emergency department admission: patient assessed by IBD Nurse (with registrar/consultant input) as requiring admission and review within the emergency department and admitted through the Emergency Department (ED)...  The ED notified Gastroenterology team on patient's arrival, who then attended ED to assess/admit patient.” | Timely access to clinical evaluation & treatment  Collaborative health professional working |
| Gethins (2020), UK | Avoid attendance (admission) | Pg20 “According to the IBD Standards Group, patients with IBD tend to access healthcare guidance from either their GP or an IBD clinical nurse specialist. Specialist nurses play a pivotal role in IBD services, including providing intervention during exacerbations of disease activity, thereby reducing the need for face-to-face consultations. By making direct contact with patients having an exacerbation to facilitate direct admission or avoid attendance…  …as appropriate, specialist IBD teams are able to collaborate with the emergency department to streamline admissions” | Timely access to clinical advice about symptoms  Timely access to clinical evaluation & treatment  Collaborative health professional working |
|  | Avoid admission | Pg24 “Analysis of the study findings suggested that the aim was to try and resolve the issues the participants were having, thereby avoiding admission. However, despite intervention, some patients still required acute access for treatment.” | Timely access to clinical advice (resolving issues) |
|  | Potentially avoided | Pg25 “The lack of urgent face-to face review resulted in admissions that could have potentially been avoided.”  Pg24 “Admission could have potentially been avoided for the 16% of participants who were not initiated on any treatment linked with the management of an acute exacerbation of IBD” | Need for face-to-face reviews  Timely access to clinical evaluation & treatment |
| Sack et al. (2012), Australia | Reducing need for inpatient care | Pg309 “As IBD is a chronic disease and it is difficult to tailor care  contemporaneously to patients' needs when they are only seen episodically, it is highly likely that methods (such as ours) which enhance their access to timely clinical advice/ contact (and include more routine contacts by nurse, phone, email) will improve clinical outcomes and reduce the need for inpatient care.” | Timely access to clinical advice |
| Martinez-Vinson et al. (2020), France | Reducing hospital admissions | Pg4 “This study suggests that introducing an IBD nurse position in a tertiary centre changes the lines, reinforces the IBD team and thus reduces hospital admissions….  Instead of the traditional model, the IBD nurse provides accessible advice and allows the patients to be outpatients.” | Collaborative health professional working  Timely access to clinical advice |
| Karimi et al (2021), Australia | Avoidance of hospital admissions, ED visits | Pge773 “one hospital admission were avoided as a result of the nurse-led advice line consultations…Advice line consults also resulted in six avoided ED presentations and one avoided hospital admission”  Pge774 “Of the 58 ED presentations avoided, 26 were avoided as IBD outpatient department appointments were expedited...  Another 26 were avoided because patients were instructed by the IBD nurse to present to their GP instead. In five instances, the patients were instructed to present to the IBD outpatient department and in one instance the matter was fully addressed through the virtual clinic thus avoiding ED and GP consultation.” | Timely access to clinical advice  Rapid access to outpatient care |
|  | Reduction in ED presentations | Pge775 “the considerable reductions in the number of ED representations as well as IBD OPD visits as the result of the implementation of the virtual clinic is arguably an indication of improved quality of life and disease control. The process of triaging patients within the structure of the virtual clinic, helped identify those patients who needed urgent care in a timely manner. In addition, the advice line enabled timely response to the patients’ psychosocial needs and concerns, contributing to patient quality of life.” | Timely access to clinical evaluation & treatment  Timely access to clinical advice (psychosocial needs) |
| IBD UK 2021, UK | Potentially avoidable flares and hospital admissions | Pg11 “Problem: People with IBD are not able to get specialist treatment and care quickly enough. This is resulting in high numbers of emergency hospital admissions and potentially avoidable flares, where their condition is not well-controlled and serious complications can arise.” | Access to specialist clinical evaluation & treatment |
|  | Emergency admissions | Pg44 “Emergency admissions and emergency surgery are often a result of missed opportunities for earlier treatment.” | Missed opportunities for earlier treatment |
|  | Reduce admissions | Pg29 “Delivered well, personalised care has positive effects on patients’ decision making, levels of confidence and ability to manage a health condition. It has been shown to reduce admissions and contact with the NHS.” | Personalised care |
| Goren et al. (2022), Israel | Frequent ED visits and hospitalisations | Pg148 “In fact, ED visits and hospitalizations are considered indicators of complicated CD…  However, frequent ED visits may also serve as a surrogate marker for a failure in inpatient IBD care, because of suboptimal disease management…  lack of accessibility to medical and support, and/or poor patient compliance” | Indicate complicated disease  A failure of outpatient care  Access issues |
|  | Unplanned ED visits | Pg 151 “These unplanned ED visits imply that IBD care may be limited by suboptimal disease control, nonspecialist care, or non-compliance with treatment” | Suboptimal quality of care |
| Hassid et al. (2023), USA | Reducing recurrent emergency care utilization | Pg29 “We hypothesized that prompt outpatient follow-up would be associated with a lower risk of recurrent ED utilization for IBD flares” | Prompt outpatient follow up |
